# Epigenetic Alterations Beyond CpG Islands in Periodontitis: In Silico Study of DNA Methylation Data

**DOI:** 10.1002/cre2.70312

**Published:** 2026-03-12

**Authors:** Koki Yoshida, Gowri Sivaramakrishnan, Farah Asa'ad

**Affiliations:** ^1^ Division of Oral Medicine and Pathology, Department of Human Biology and Pathophysiology, School of Dentistry Health Sciences University of Hokkaido Hokkaido Japan; ^2^ Bahrain Defence Force Royal Medical Services Riffa Bahrain; ^3^ Department of Oral Biochemistry, Institute of Odontology, Sahlgrenska Academy University of Gothenburg Gothenburg Sweden

**Keywords:** CpG islands, DNA methylation, epigenetics, Gene Ontology analysis, methylation directionality, periodontitis

## Abstract

**Objectives:**

To classify differentially methylated CpG sites in periodontitis based on methylation shift direction and location relative to CpG islands.

**Material and Methods:**

We integrated DNA methylation data from 65 gingival samples (31 periodontitis, 34 healthy) from three GEO datasets. Preprocessing included normalization using R pipelines. Differential DNA methylation analysis was performed with the limma package. CpG sites (adjusted *p* < 0.05) were further evaluated using an exploratory threshold‐crossing framework based on group‐mean *β*‐values (cutoff = 0.5) identifying Control low → Periodontitis high and Control high → Periodontitis low shifts, annotated with Illumina 450k references. Top 20 CpG‐associated genes were selected by absolute logFC. Gene Ontology (GO) enrichment was conducted using clusterProfiler.

**Results:**

We identified 164,400 differentially methylated CpG sites, including CpGs exhibiting Control low → Periodontitis high and Control high → Periodontitis low shifts, and mapped to gene features. Nearly half of methylation alterations (47.77%) were in OpenSea regions, with additional signals in CpG islands and promoter‐proximal regions. GO enrichment analysis revealed that differentially methylated genes in periodontitis were associated with epithelial and organ morphogenesis, including epithelial tube morphogenesis, epithelial cell proliferation, and cell fate commitment. Enriched cellular components were membrane‐ and vesicle‐associated structures, while molecular functions were transmembrane transporter activity, ion channel activity, and DNA‐binding transcription factor–related functions. These findings suggest that DNA methylation alterations in periodontitis are linked to epigenetic regulation of epithelial structure and tissue remodeling, with implications for immune‐related cellular processes.

Among the top 20 genes exhibiting β‐threshold–based directionality changes, 13 were significantly enriched in GO terms: 8 in the Control low → Periodontitis high group (*DGKA*, *CHMP6*, *RUNX2*, *CDKL1*, *SLC6A3*, *CFTR*, *HLA‐C*, *LRIG1*), 5 in the Control high → Periodontitis low group (*AP2A2*, *PARD3*, *PADI2*, *FOXK1*, *RGS1*).

**Conclusions:**

This study provides an exploratory, direction‐aware summary framework of DNA methylation in periodontitis, highlighting functional changes beyond CpG islands and identifying candidate genes for further functional validation.

**Abbreviations**
AP2A2: Adaptor Related Protein Complex 2 Subunit Alpha 2
BP: Biological Process
CAL: Clinical Attachment Loss
CC: Cellular Component
CDKL1: Cyclin Dependent Kinase Like 1
CFTR: CF Transmembrane Conductance Regulator
CHMP6: Charged Multivesicular Body Protein 6
DGKA: Diacylglycerol Kinase Alpha
EWAS: Epigenome‐Wide Association Studies
FDR: False Discovery Rate
FOXK1: Forkhead Box K1
GEO: Gene Expression Omnibus
GO: Gene Ontology
LPS: Lipopolysaccharide
LRIG1: Leucine Rich Repeats and Immunoglobulin Like Domains 1
MF: Molecular Function
PADI2: Peptidyl Arginine Deiminase 2
PARD3: Par‐3 Family Cell Polarity Regulator
PCA: Principal Component Analysis
RGS1: Regulator of G Protein Signaling 1
RUNX2: RUNX Family Transcription Factor 2
SLC6A3: Solute Carrier Family 6 Member 3
SYMBOL: Annotated Gene Symbols
TSS: Transcription Start Site
UCSC: University of California Santa Cruz
UTR: Untranslated Region

## 1 Introduction

Periodontitis is a common chronic inflammatory disease characterized by progressive destruction of the periodontal ligament and alveolar bone, ultimately leading to tooth loss (Kinane et al. 2017). It arises from dysbiosis of the oral microbiome and an exaggerated or dysregulated host immune response (Bartold and Van Dyke 2013). While conventional research has highlighted the contributions of microbial, environmental, and genetic factors, increasing attention has turned toward epigenetic regulation, particularly DNA methylation, as a key mechanism modulating host responses in periodontitis pathogenesis (Barros et al. 2018).

Several previous epigenome‐wide association studies (EWAS) have examined gingival tissue, peripheral blood, and immune cells in the context of periodontitis (reviewed in Cho et al. 2021; reviewed in Suzuki and Yamada 2022; Zhao et al. 2023). However, most of these studies focused on identifying differences in methylation levels between healthy and diseased tissues, without clarifying whether CpG sites exhibit a threshold‐crossing shift between low and high methylation states (*β* cutoff = 0.5) at the group level. To our knowledge, no previous study has systematically investigated this “directionality of change” in DNA methylation during the onset of periodontitis, particularly focusing on CpG sites that exhibit a threshold‐crossing shift between low and high methylation states at the group level. Furthermore, previous studies have used inconsistent criteria for defining hypermethylation, often based on arbitrary thresholds derived from *β*‐values, with no standardized evaluation method established to date.

To address this limitation, we adopted the classification approach described by Alholle et al. (2015) where CpG sites with methylation levels ≥ 50% are defined as hypermethylated, and those with < 50% as hypomethylated. Based on this definition, we first determined the hypermethylation or hypomethylation status for each CpG site in both healthy and periodontitis tissues (Alholle et al. 2015). To clearly distinguish between methylation state and direction of change, we hereafter refer to CpG sites with *β* ≥ 0.5 and *β* < 0.5 as “high‐methylation” and “low‐methylation” states, respectively. We then focused specifically on CpG sites that cross this threshold between groups (Control low → Periodontitis high or Control high → Periodontitis low) as an exploratory, group‐level summary of methylation directionality.

Another notable feature of our approach is the spatial genomic annotation of each CpG site. We classified CpGs by their relation to CpG islands (Island, Shore, Shelf, OpenSea), as well as to gene structural elements (TSS200, TSS1500, 5′ UTR, 1stExon, Gene body, 3′ UTR) (Jones 2012). This spatial stratification enabled us to map methylation changes not only to promoter‐proximal regions but also to distal regulatory elements and intragenic regions, which are increasingly recognized as important for transcriptional fine‐tuning. There remains a lack of integrated approaches that simultaneously consider the spatial relationship of CpG regions and gene structural domains in large‐scale DNA methylation studies of inflammatory conditions. This analytical gap is particularly evident in the context of chronic inflammatory diseases such as periodontitis, where no previous studies have applied such classification systematically. Our study addresses this need by providing a genome‐wide cross‐tabulation framework that captures both spatial and structural dimensions of methylation change.

Furthermore, we separately analyzed hypermethylated and hypomethylated CpG sites and linked them to associated genes. From these, we conducted GO enrichment analysis on the genes, providing a comprehensive landscape of the functional pathways potentially regulated by differential DNA methylation in periodontitis.

This study provides a systematic and spatially contextualized epigenetic profile of periodontitis, offering new insights into its regulatory architecture and biological implications.

## 2 Materials and Methods

### 2.1 Data Collection and Integration

Publicly available DNA methylation datasets were obtained from the Gene Expression Omnibus (GEO) under the accession numbers GSE53849, GSE59932, and GSE59939 (De Souza et al. 2014; Planello et al. 2016). GSE59932 and GSE59939 represent sub‐series of the same study (SuperSeries GSE59962) and therefore share a common primary publication (Planello et al. 2016). These datasets included a total of 34 healthy control samples and 31 periodontitis samples, all analyzed using the Illumina HumanMethylation450 BeadChip platform. Because all datasets used the same array platform and were derived from gingival tissue collected under comparable protocols from the same institution (Piracicaba Dental School, University of Campinas, UNICAMP, Brazil), platform‐ and site‐related heterogeneity was minimized, supporting integrated analysis. The sample distribution was as follows: GSE53849 included 11 control and 12 periodontitis samples; GSE59932 included 12 control and 10 periodontitis samples; and GSE59939 included 11 control and 9 periodontitis samples. Raw methylation data (*β*‐values) were downloaded and processed using R (version 4.3.0). All subsequent preprocessing harmonization, statistical analyses, and downstream data generation reported in this study were conducted using R (version 4.5.0) to ensure reproducibility and consistency across datasets.

GSE53849 included samples from 12 chronic periodontitis patients and 11 age‐ and gender‐matched healthy controls, with mean ages of 50.63 and 50.42 years, respectively (De Souza et al. 2014). Chronic periodontitis was diagnosed based on clinical attachment loss (CAL ≥ 5 mm) and bleeding on probing in at least one site. Control samples were obtained from non‐inflamed gingival areas in patients undergoing surgery for non‐periodontal reasons, showing no signs of inflammation or CAL > 3.5 mm. Biopsies (~2 × 2 mm) included junctional epithelium and soft connective tissue. Exclusion criteria included smoking, systemic diseases affecting periodontal health, recent use of antibiotics or anti‐inflammatory drugs, pregnancy, and lactation.

GSE59932 and GSE59939 included biopsies from a total of 19 chronic periodontitis patients and 23 healthy controls (Planello et al. 2016). Age information was not reported for these datasets. Participants met similar exclusion criteria, including the absence of smoking, systemic disease, recent medication, pregnancy, lactation, and alcohol use. Samples were taken from a single tooth and included junctional epithelium and connective tissue. Control tissues were collected during aesthetic procedures or third molar extractions, and showed no clinical signs of periodontitis.

Ethnicity was not explicitly reported in the original studies.

### 2.2 Data Preprocessing and Cleaning

The three datasets were merged into a unified CpG‐by‐sample matrix using common probe IDs. Quality control and preprocessing were conducted using the minfi, wateRmelon, and data.table packages in R. CpG sites with more than 80% missing *β*‐values across samples were removed, resulting in a final set of 484,331 CpG sites retained for downstream analyses, while no samples were excluded. Sample metadata was aligned and integrated with the beta matrix to ensure consistent labeling across samples. All preprocessing steps were applied uniformly to all samples using a single, reproducible analytical pipeline.

### 2.3 Differential Methylation Analysis and Data Transformation

Differential methylation results comparing periodontitis and healthy control groups were obtained using the limma‐based analyses of publicly available GEO data. After quality control and probe filtering as described in Section 2.2, these differential methylation result tables derived from publicly available GEO data were used as input for all downstream analyses.

*β*‐values were logit‐transformed to M‐values prior to statistical modeling, as M‐values provide improved statistical properties for differential methylation analysis.

A design matrix representing disease status (periodontitis vs. control) was constructed, and linear models were fitted for each CpG site using the lmFit() function. Empirical Bayes moderation was applied using the eBayes() function to stabilize variance estimates across CpG sites in this high‐dimensional dataset.

Significantly differentially methylated CpG sites were defined as those with an adjusted *p*‐value < 0.05 based on the Benjamini–Hochberg false discovery rate (FDR), a threshold widely used in high‐dimensional genomic analyses employing limma and empirical Bayes methods (Ritchie et al. 2015; Phipson et al. 2016).

The resulting differential methylation statistics, including log fold change (logFC), moderated t‐statistics, raw *p*‐values, adjusted *p*‐values, and B‐statistics (the log‐odds of differential methylation as defined in the limma framework), were obtained for all 484,331 CpG sites and are provided in Supplementary File 1, together with the corresponding R script and README to ensure full reproducibility. These files represent the finalized outputs of the limma‐based analysis used throughout the manuscript.

### 2.4 CpG Annotation and Gene Mapping

Significant CpG sites were annotated to genes using the IlluminaHumanMethylation450kanno.ilmn12.hg19 annotation package in R (version ≥ 4.3.0). Annotations included gene symbols, genomic coordinates, and CpG island context. The mapping was performed by merging CpG probe IDs with the official annotation data provided in the Bioconductor package. In cases where a probe mapped to multiple genes, all associated mappings were retained to preserve biological interpretation. The resulting annotated dataset is provided in Supplementary File 2. This annotation represents the finalized mapping used for all downstream analyses and tables reported in this study. The accompanying R script and instructions used for the annotation are included in Supplementary File 2, while the Illumina 450K annotation reference table together with its export script and README are provided in Supplementary File 3.

### 2.5 Differential Methylation Visualization Using a Volcano Plot

Differential methylation results obtained as described in Section 2.3 were visualized using a volcano plot.

The volcano plot was generated using the ggplot2 package (version 3.4.4) in R. LogFC values derived from the limma analysis were plotted on the x‐axis, and −log10‐transformed Benjamini–Hochberg adjusted *p*‐values were plotted on the y‐axis.

**Figure 1:**
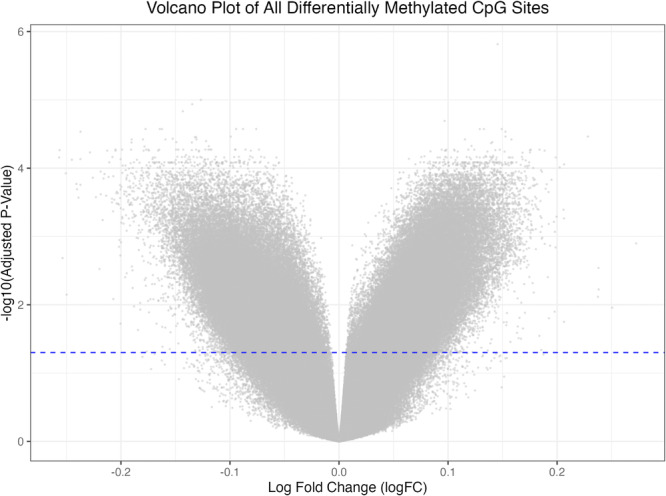
Volcano plot of all differentially methylated CpG sites. This volcano plot summarizes differential DNA methylation results across 484,331 CpG sites analyzed by limma. The x‐axis represents the log fold change (logFC) between periodontitis and control groups, and the y‐axis shows the −log10 adjusted *p*‐value based on Benjamini–Hochberg correction. Non‐significant CpGs are densely concentrated near the center, while CpG sites with larger effect sizes and statistical significance (adjusted *p* < 0.05) are distributed toward both tails. The horizontal dashed line indicates the statistical significance cutoff (adjusted *p* = 0.05). Vertical logFC lines were omitted. The figure was generated using the *ggplot2* package in R (version 4.5.0).

All 484,331 CpG sites analyzed were included in the plot, regardless of statistical significance. A dashed horizontal line corresponding to −log10(0.05), which is approximately 1.301 was added to illustrate the conventional adjusted *p*‐value threshold. Vertical logFC threshold lines were intentionally omitted to emphasize statistical significance rather than effect size cutoffs. The resulting volcano plot is shown in Figure 1. The R script and README used to generate Figure 1 (Volcano Plot) are provided in Supplementary_Figure_1_VolcanoPlot_script.R and Supplementary_Figure_1_VolcanoPlot_README.txt.

### 2.6 Hierarchical Clustering and Heatmap Visualization

**Figure 2:**
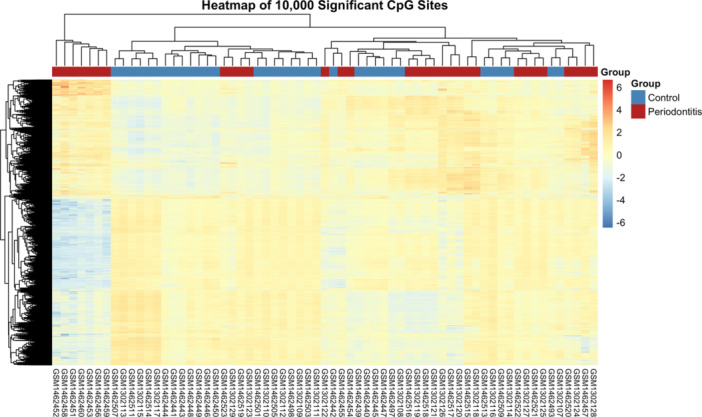
Heatmap of 10,000 significantly differentially methylated CpG sites. This heatmap shows hierarchical clustering based on Z‐score normalized *β*‐values across samples for 10,000 randomly sampled significantly differentially methylated CpG sites (adjusted *p* < 0.05) across 65 gingival tissue samples (31 periodontitis and 34 control samples). Each row represents a CpG site, and each column represents a sample. Control and periodontitis samples are annotated in blue and red, respectively, above the heatmap. Methylation levels are color‐coded (blue = lower, red = higher). The figure reveals distinct clustering patterns, with samples from the same group tending to cluster together, indicating group‐specific methylation profiles associated with periodontitis.

*β*‐Values for all samples were compiled from three GEO datasets (GSE53849, GSE59932, and GSE59939) to construct a CpG‐by‐sample methylation matrix. From the full set of 484,331 CpG sites, a subset of 164,400 significantly differentially methylated CpGs (adjusted *p* < 0.05) was selected. A fixed random seed was set to ensure reproducibility, and 10,000 CpGs were randomly sampled from this significant subset to visualize global methylation patterns. *β*‐values were transformed to Z‐scores across samples to enable clustering. Hierarchical clustering of both CpGs and samples was performed using Euclidean distance and complete linkage. Sample group annotations (Control vs. Periodontitis) were added to the column side of the heatmap and color‐coded (blue for Control, red for Periodontitis). The heatmap was generated using the pheatmap package (version 1.0.12) and is shown in Figure 2. The Z‐score matrix and sample metadata used to generate this heatmap are provided in Supplementary File 4 and Supplementary File 5, respectively. The R script and README used to generate Figure 2 are provided in Supplementary_Figure_2_Heatmap_script.R and Supplementary_Figure_2_Heatmap_README.txt.

### 2.7 Sample Clustering and Dimensionality Reduction

**Figure 3:**
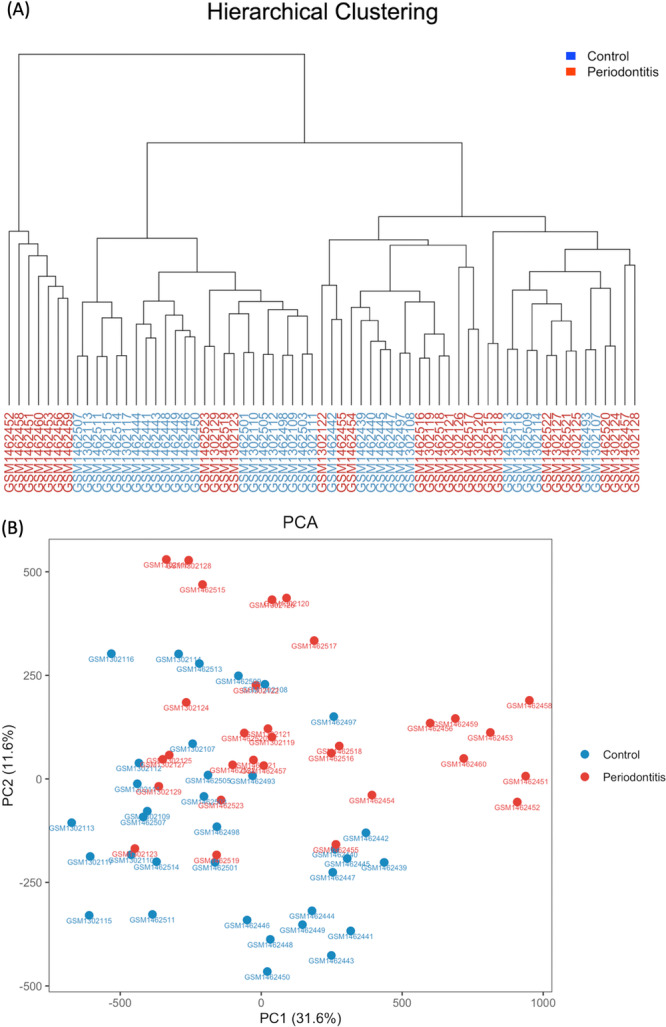
Unsupervised analyses based on the randomly sampled 10,000 significant CpG sites. (A) Hierarchical clustering dendrogram of 65 samples (31 periodontitis, 34 control) based on Euclidean distances of Z‐score normalized *β*‐values from 10,000 randomly sampled significantly differentially methylated CpG sites (adjusted *p* < 0.05). Samples are color‐coded by group (red: periodontitis, blue: control). The clustering clearly separates the two groups, supporting consistent methylation differences aligned with clinical status. (B) Principal component analysis (PCA) plot of the first two components, PC1 (31.6%) and PC2 (11.6%), derived from the same CpG subset. Each point represents a sample, color‐coded by group. The PCA further supports group separation, with distinct distributions between control and periodontitis samples, demonstrating that the main sources of methylation variance correspond to disease status.

Unsupervised hierarchical clustering and dimensionality reduction analyses were conducted using 10,000 randomly sampled differentially methylated CpG sites. Z‐score normalization was applied across samples for each CpG site. Hierarchical clustering was performed using Euclidean distance and complete linkage. The dendrogram was visualized using the dendextend package (version 1.19.0), with sample labels colored by group. Principal component analysis (PCA) was conducted using the prcomp() function (stats package). Data were Z‐score normalized per CpG site prior to PCA; therefore, additional scaling was not applied. The first two principal components were visualized in a scatter plot, with sample groups indicated by color. The same Z‐score matrix and sample metadata described in Section 2.6 (Supplementary Files 4 and 5) were also used in this analysis. The R script and README used to generate Figure 3 (Hierarchical Clustering and PCA) are provided in Supplementary_Figure_3_Hierarchical clustering and PCA_script.R and Supplementary_Figure_3_Hierarchical clustering and PCA_README.txt.

### 2.8 Directionality Classification and Cross‐Tabulation Analysis

Group‐wise average *β*‐values were calculated separately for control and periodontitis samples for each CpG site. CpG sites were assigned a “high‐methylation” state (*β* ≥ 0.5) or “low‐methylation” state (*β* < 0.5) based on group‐wise mean *β*‐values, following the thresholding concept used by Alholle et al. (2015).

For clarity, these thresholds are hereafter referred to as high‐methylation (*β* ≥ 0.5) and low‐methylation (*β* < 0.5) states, respectively, to distinguish methylation state from direction of change.

When used in the context of directionality, the terms “Control low → Periodontitis high” and “Control high → Periodontitis low” refer specifically to threshold‐crossing changes between groups, namely Control low (*β* < 0.5) → Periodontitis high (*β* ≥ 0.5) and Control high (*β* ≥ 0.5) → Periodontitis low (*β* < 0.5), respectively, rather than simply higher or lower methylation levels in the disease group.

This classification scheme was applied as an exploratory framework to summarize group‐level methylation trends and was not intended to replace continuous differential methylation analysis based on linear modeling. The fixed *β*‐value threshold (*β* ≥ 0.5 for high methylation and *β* < 0.5 for low methylation) was adopted following the approach described by Alholle et al. (2015).

From CpG sites classified into either “Control low → Periodontitis high” or “Control high → Periodontitis low” categories, the top 20 genes with the largest absolute log fold change (logFC) were extracted (Table 1). Genomic spatial annotations relative to CpG islands (Relation_to_Island) and gene structural regions (UCSC_RefGene_Group) were assigned using Illumina HumanMethylation450K annotation data. CpG sites lacking annotation were categorized as “unknown.”

**Table 1:** Top 20 genes with the largest methylation changes between control and periodontitis groups.

Top 20 genes						
Methylation change direction	Gene symbol	Mean logFC	Mean β in control	Mean β in periodontitis	CpG IDs	RefSeq IDs
Control low → Periodontitis high (*β* cutoff = 0.5)	SCARA3	0.206323466	0.325088974	0.550743732	cg27024127	NM_182826;NM_016240
	DGKA	0.186103880	0.463929373	0.636142842	cg07679948	NM_201445;NM_201554;NM_001345;NM_201444
	CHMP6	0.180451113	0.427431010	0.594948176	cg17714010	NM_024591
	GRAP2	0.176343218	0.345254507	0.503365491	cg17988310	NM_004810
	ANP32A	0.174294498	0.487796838	0.645383927	cg16068038	NM_006305
	C15orf28	0.174294498	0.487796838	0.645383927	cg16068038	NR_026808
	RUNX2	0.173846695	0.353517831	0.511252409	cg23261343	NM_001015051;NM_001024630;NM_004348
	FAM117A	0.171448251	0.476391397	0.648757545	cg18824549	NM_030802
	HADHA	0.170528506	0.382071673	0.551263074	cg01188578	NM_000182
	CDKL1	0.170304259	0.347179142	0.506939328	cg19930116	NM_004196
	LRP12	0.170284430	0.343858054	0.513716775	cg04104738	NM_001135703;NM_013437
	SLC6A3	0.170098885	0.441638466	0.586061634	cg21163347	NM_001044
	C12orf68	0.168545035	0.386249035	0.541225053	cg15959252	NM_001013635
	FAM113B	0.168034390	0.435914187	0.606991496	cg06547285	NM_138371
	CFTR	0.166829935	0.346429017	0.511778447	cg09181792	NM_000492
	HLA‐C	0.165545061	0.360388834	0.527175698	cg01521131	NM_002117
	LRIG1	0.163155815	0.401644742	0.554800577	cg02012974	NM_015541
	DENND3	0.162320098	0.402965665	0.543888173	cg10113526	NM_014957
	PITPNC1	0.161855428	0.366411863	0.520185872	cg02018291	NM_181671;NM_012417
	RTEL1	0.161301355	0.424989511	0.558145266	cg11012153	NM_016434;NM_032957
Control high → Periodontitis low (*β* cutoff = 0.5)	TUBA3E	−0.253582979	0.585531722	0.356951427	cg03206401	NM_207312
	ATP11A	−0.249858039	0.555195536	0.337820462	cg21463262	NM_015205;NM_032189
	PIGR	−0.245055045	0.566022491	0.338099343	cg09763644	NM_002644
	CLPTM1L	−0.220941109	0.600675563	0.383382931	cg05905124;cg07493874;cg13325231;cg14733637	NM_030782
	ZCCHC14	−0.212582756	0.625815745	0.415511888	cg06545761	NM_015144
	AP2A2	−0.200483242	0.620273306	0.403857224	cg16999994	NM_012305
	PARD3	−0.200458597	0.671931754	0.467683729	cg00287536	NM_019619
	LOC100310782	−0.196206215	0.690141317	0.495179435	cg08238319	NR_029397
	LOC283663	−0.194987713	0.600691974	0.415414743	cg13626582	NR_024433
	HSBP1L1	−0.193501967	0.591994938	0.409595168	cg16313807	NM_001136180
	PADI2	−0.192135567	0.635289822	0.451695586	cg00569276	NM_007365
	DNASE1L3	−0.191755698	0.593708209	0.416646038	cg24163658	NM_004944
	FOXK1	−0.190894875	0.637544942	0.456298977	cg04261496	NM_001037165
	KCTD5	−0.190602181	0.545845151	0.366788453	cg08055663	NM_018992
	RHBDF2	−0.189237826	0.529895316	0.362415650	cg20690125	NM_001005498;NM_024599
	LOC100129637	−0.189079286	0.550352070	0.381558926	cg00270654;cg00227665;cg05304979;cg07041681;cg07102001;cg07396047;cg07721872;cg09276655;cg09331545	NR_024488
	DMWD	−0.188083011	0.656464140	0.469728042	cg03758011	NM_004943
	BCL2L14	−0.187658640	0.590237614	0.412063339	cg20141578	NM_138722;NM_138723;NM_030766
	RGS1	−0.186322336	0.663882933	0.486950604	cg10861751	NM_002922
	TRAF5	−0.184529767	0.552903195	0.385134487	cg13066703	NM_004619;NM_145759;NM_001033910

*Note:* This table summarizes the top 20 genes associated with the largest absolute methylation differences (|logFC|) among significantly differentially methylated CpG sites (adjusted *p*‐value < 0.05, limma). CpG sites were grouped by threshold‐crossing directionality using group‐wise mean *β*‐values with a cutoff of 0.5: “Control low (*β* < 0.5) → Periodontitis high (*β* ≥ 0.5)” and “Control high (*β* ≥ 0.5) → Periodontitis low (*β* < 0.5).” Genes were ranked by the largest absolute log fold change (|logFC|) observed among CpG sites within these threshold‐crossing categories. Each entry reports the corresponding CpG ID(s), CpG island context (Island, Shore, Shelf, OpenSea), group‐wise mean *β*‐values in both groups, and RefSeq IDs. This exploratory summary highlights candidate genes showing clear between‐group shifts across the *β* = 0.5 threshold.

Stratified cross‐tabulations integrating methylation directionality, CpG island–defined regions, and gene structural regions were generated for CpG sites mapped to these top 20 genes and summarized in Table 2.

**Table 2:** Stratified classification of differentially methylated CpG sites in top‐ranked genes associated with periodontitis.

Relation_to_Island	UCSC_RefGene_Group	Methylation state (*β* cutoff = 0.5)	Percentage of CpG_count of methylation state group in total (%)	Methylation_Status_Change (high; *β* ≥ 0.5, low; *β* < 0.5)	Percentage of CpG_count of Meth_Status_Change in total (%)	CpG_count in Meth_Status_Change	UCSC_RefGene_Name
Island (19.86%)	TSS200	High‐methylation	1.10	Control low → Periodontitis high	0.02	63	
		Low‐methylation	0.08	Control high → Periodontitis low	0.01	14	*TUBA3E*
	TSS1500	High‐methylation	0.94	Control low → Periodontitis high	0.03	80	
		Low‐methylation	0.07	Control high → Periodontitis low	< 0.01	12	
	5'UTR	High‐methylation	0.42	Control low → Periodontitis high	0.02	49	*DGKA*
		Low‐methylation	0.05	Control high → Periodontitis low	< 0.01	4	
	1stExon	High‐methylation	0.41	Control low → Periodontitis high	0.02	46	
		Low‐methylation	0.05	Control high → Periodontitis low	< 0.01	7	
	Body	High‐methylation	1.63	Control low → Periodontitis high	0.11	302	
		Low‐methylation	0.68	Control high → Periodontitis low	0.03	86	
	3'UTR	High‐methylation	0.09	Control low → Periodontitis high	0.01	20	
		Low‐methylation	0.04	Control high → Periodontitis low	< 0.01	8	*ZCCHC14*
	Unknown	High‐methylation	11.83	Control low → Periodontitis high	0.12	313	
		Low‐methylation	2.48	Control high → Periodontitis low	0.05	119	
N_Shore (12.60%)	TSS200	High‐methylation	0.26	Control low → Periodontitis high	0.01	34	
		Low‐methylation	0.05	Control high → Periodontitis low	< 0.01	10	
	TSS1500	High‐methylation	0.78	Control low → Periodontitis high	0.06	160	
		Low‐methylation	0.50	Control high → Periodontitis low	0.03	73	*HSBP1L1*
	5'UTR	High‐methylation	0.19	Control low → Periodontitis high	0.02	54	
		Low‐methylation	0.11	Control high → Periodontitis low	0.01	16	
	1stExon	High‐methylation	0.03	Control low → Periodontitis high	< 0.01	4	
		Low‐methylation	0.02	Control high → Periodontitis low	< 0.01	2	
	Body	High‐methylation	0.67	Control low → Periodontitis high	0.08	200	*HLA‐C*
							*LRP12*
		Low‐methylation	0.80	Control high → Periodontitis low	0.04	105	*DMWD*
							*LOC100129637*
	3'UTR	High‐methylation	0.06	Control low → Periodontitis high	0.01	21	
		Low‐methylation	0.07	Control high → Periodontitis low	< 0.01	8	*ATP11A*
	Unknown	High‐methylation	5.34	Control low → Periodontitis high	0.07	193	
		Low‐methylation	3.72	Control high → Periodontitis low	0.05	143	
S_Shore (9.80%)	TSS200	High‐methylation	0.24	Control low → Periodontitis high	0.02	43	
		Low‐methylation	0.05	Control high → Periodontitis low	< 0.01	10	
	TSS1500	High‐methylation	0.67	Control low → Periodontitis high	0.06	167	
		Low‐methylation	0.46	Control high → Periodontitis low	0.03	71	*PADI2*
	5'UTR	High‐methylation	0.16	Control low → Periodontitis high	0.02	43	
		Low‐methylation	0.08	Control high → Periodontitis low	< 0.01	11	
	1stExon	High‐methylation	0.02	Control low → Periodontitis high	< 0.01	9	*C12orf68*
		Low‐methylation	0.01	Control high → Periodontitis low	< 0.01	3	
	Body	High‐methylation	0.62	Control low → Periodontitis high	0.06	166	*CHMP6*
		Low‐methylation	0.53	Control high → Periodontitis low	0.03	74	*RHBDF2*
	3'UTR	High‐methylation	0.04	Control low → Periodontitis high	0.01	15	*C12orf68*
				Control low → Periodontitis high	0.01	15	*FAM113B*
		Low‐methylation	0.06	Control high → Periodontitis low	< 0.01	8	
	Unknown	High‐methylation	4.04	Control low → Periodontitis high	0.05	135	
		Low‐methylation	2.82	Control high → Periodontitis low	0.04	118	
N_Shelf (5.20%)	TSS200	High‐methylation	0.02	Control low → Periodontitis high	< 0.01	10	
		Low‐methylation	0.03	Control high → Periodontitis low	< 0.01	7	
	TSS1500	High‐methylation	0.03	Control low → Periodontitis high	< 0.01	5	
		Low‐methylation	0.06	Control high → Periodontitis low	< 0.01	8	
	5'UTR	High‐methylation	0.03	Control low → Periodontitis high	< 0.01	11	
		Low‐methylation	0.16	Control high → Periodontitis low	0.01	16	
	1stExon	High‐methylation	0.00	Control low → Periodontitis high	< 0.01	1	
		Low‐methylation	0.00	Control high → Periodontitis low	< 0.01	1	
	Body	High‐methylation	0.15	Control low → Periodontitis high	0.02	43	*HADHA*
				Control low → Periodontitis high	0.02	43	*RTEL1*
		Low‐methylation	0.78	Control high → Periodontitis low	0.02	59	*CLPTM1L*
	3'UTR	High‐methylation	0.02	Control low → Periodontitis high	< 0.01	10	
		Low‐methylation	0.06	Control high → Periodontitis low	< 0.01	3	
	Unknown	High‐methylation	0.83	Control low → Periodontitis high	0.04	94	
		Low‐methylation	3.03	Control high → Periodontitis low	0.04	117	
S_Shelf (4.77%)	TSS200	High‐methylation	0.01	Control low → Periodontitis high	< 0.01	5	
		Low‐methylation	0.03	Control high → Periodontitis low	< 0.01	8	
	TSS1500	High‐methylation	0.02	Control low → Periodontitis high	< 0.01	10	
		Low‐methylation	0.06	Control high → Periodontitis low	< 0.01	7	
	5'UTR	High‐methylation	0.03	Control low → Periodontitis high	< 0.01	10	
		Low‐methylation	0.16	Control high → Periodontitis low	0.01	15	
	1stExon	High‐methylation	0.00	Not applicable	< 0.01	3	
		Low‐methylation	0.01	Not applicable	0.01	20	
	Body	High‐methylation	0.13	Control low → Periodontitis high	0.02	46	
		Low‐methylation	0.72	Control high → Periodontitis low	0.02	55	
	3'UTR	High‐methylation	0.01	Control low → Periodontitis high	< 0.01	4	
		Low‐methylation	0.05	Control high → Periodontitis low	< 0.01	2	
	Unknown	High‐methylation	0.62	Control low → Periodontitis high	0.03	73	
		Low‐methylation	2.93	Control high → Periodontitis low	0.04	117	
OpenSea (47.77%)	TSS200	High‐methylation	0.36	Control low → Periodontitis high	0.05	119	
		Low‐methylation	0.69	Control high → Periodontitis low	0.05	122	*LOC100310782*
				Control high → Periodontitis low	0.05	122	*RGS1*
	TSS1500	High‐methylation	0.28	Control low → Periodontitis high	0.05	120	*CFTR*
				Control low → Periodontitis high	0.05	120	*C15orf28*
		Low‐methylation	1.49	Control high → Periodontitis low	0.06	162	*LOC283663*
	5'UTR	High‐methylation	0.22	Control low → Periodontitis high	0.04	101	
		Low‐methylation	0.74	Control high → Periodontitis low	0.03	88	*BCL2L14*
	1stExon	High‐methylation	0.04	Control low → Periodontitis high	< 0.01	10	
		Low‐methylation	0.23	Control high → Periodontitis low	0.01	27	
	Body	High‐methylation	2.42	Control low → Periodontitis high	0.20	516	*ANP32A*
							*CDKL1*
							*DENND3*
							*FAM117A*
							*GRAP2*
							*LRIG1*
							*PITPNC1*
							*RUNX2*
							*SCARA3*
		Low‐methylation	6.48	Control high → Periodontitis low	0.16	413	*AP2A2*
				Control high → Periodontitis low			*DNASE1L3*
				Control high → Periodontitis low			*FOXK1*
				Control high → Periodontitis low			*KCTD5*
				Control high → Periodontitis low			*PARD3*
				Control high → Periodontitis low			*TRAF5*
	3'UTR	High‐methylation	0.14	Control low → Periodontitis high	0.02	44	*SLC6A3*
		Low‐methylation	1.04	Control high → Periodontitis low	0.02	46	*PIGR*
	Unknown	High‐methylation	8.15	Control low → Periodontitis high	0.36	946	
		Low‐methylation	25.50	Control high → Periodontitis low	0.30	785	
Unknown (0.00%)	Body	High‐methylation	0.00	Control low → Periodontitis high	< 0.01	0	
		Low‐methylation	0.00	Control high → Periodontitis low	< 0.01	0	
	Unknown	High‐methylation	0.00	Control low → Periodontitis high	< 0.01	2	

*Note:* A total of 164,400 significantly differentially methylated CpG sites (adjusted *p*‐value < 0.05; Benjamini–Hochberg FDR) were identified using limma from integrated methylation data across three GEO datasets (34 control and 31 periodontitis samples). CpG sites were classified based on threshold‐crossing directionality using group‐wise average *β*‐values and a cutoff of 0.5: “Control low → Periodontitis high” (hypermethylation shift) and “Control high → Periodontitis low” (hypomethylation shift). High‐methylation and low‐methylation indicate methylation states defined by group‐wise average *β*‐values (*β* ≥ 0.5 and *β* < 0.5, respectively), whereas directionality is specified separately by the indicated status changes. Among the top 20 genes listed in Table 1, only those corresponding to either hypermethylation or hypomethylation were retained and listed in the UCSC_RefGene_Name column in Table 2. Each row represents a CpG site and includes annotations for genomic context relative to CpG islands (Relation_to_Island: Island, Shore, Shelf, OpenSea, or unknown) and gene structure (UCSC_RefGene_Group: TSS200, TSS1500, 5′ UTR, 1stExon, Body, 3′ UTR, or unknown). Percentages are calculated using as the denominator the total number of significantly differentially methylated CpG sites mapped to the top 20 genes listed in Table 1. “Not applicable” indicates that no CpGs were observed in the corresponding stratum. This stratified presentation facilitates biologically meaningful interpretation of CpG methylation changes associated with periodontitis.

For CpG island–defined regions, Relation_to_Island categories were ordered as follows: CpG islands, north and south shores (≤ 2 kb flanking CpG islands), north and south shelves (~2–4 kb outside shores), and OpenSea regions (> 6 kb from CpG islands), according to established definitions (Shen et al. 2013). Unknown spatial relationships were grouped as “unknown.”

For gene structural annotation, UCSC_RefGene_Group categories were ordered by proximity to the transcription start site: TSS200 (within 200 bp of the transcription start site), TSS1500 (200–1500 bp upstream), 5′ untranslated region (5′ UTR), first exon (1stExon), gene body (exons and introns), and 3′ untranslated region (3′ UTR). Unknown annotations were grouped as “unknown.”

This consistent ordering facilitated interpretation of spatial methylation patterns across genomic regions and enabled systematic comparison between CpG island–proximal and distal methylation changes. All annotated CpG data, group‐wise average *β*‐values, and scripts used to generate Tables 1 and 2 are provided in Supplementary File 6.

### 2.9 Gene Ontology Enrichment Analysis

To investigate the biological implications of differential DNA methylation in periodontitis, Gene Ontology (GO) enrichment analysis was performed using the R package *clusterProfiler* (v4.6.2). A total of 164,400 significantly differentially methylated CpG sites (adjusted *p* < 0.05) were identified, and 19,605 annotated gene symbols (SYMBOL) were extracted from these CpGs. These gene symbols were converted to Entrez Gene IDs using the *org.Hs.eg.db* annotation package. GO enrichment analysis was then conducted with enrichGO() across three ontologies: Biological Process (BP), Cellular Component (CC), and Molecular Function (MF). The parameters used included: pvalueCutoff = 0.05, qvalueCutoff = 0.2, and pAdjustMethod = “BH” (Benjamini–Hochberg method). The top 20 significantly enriched GO terms were selected from each category and visualized. Furthermore, for each enriched GO term, Table 3 lists the gene symbols that were both among the top 20 differentially methylated genes and enriched in that GO term. The full dataset of enriched GO terms, along with the R script and README used to reproduce Table 3, is provided in Supplementary File 7: Table 3 Generation Script and README.

**Table 3:** Enriched GO terms derived from all significantly differentially methylated CpG sites, with annotation of relevant top 20 genes.

	Rank	ID	Description	geneID	pvalue	p.adjust	qvalue	RichFactor	FoldEnrichment	zScore	GeneRatio	BgRatio	Count
BP	1	GO:0060562	Epithelial tube morphogenesis		2.07E−23	1.32E−19	7.73E−20	0.970326409	1.236748552	8.369340506	327/14754	337/18805	327
	2	GO:0048880	Sensory system development	*SLC6A3*	2.84E−20	9.05E−17	5.31E−17	0.943661972	1.20276287	8.078508368	402/14754	426/18805	402
	3	GO:0150063	Visual system development	*SLC6A3*	1.07E−19	2.27E−16	1.33E−16	0.942720764	1.201563235	7.96294272	395/14754	419/18805	395
	4	GO:0001654	Eye development	*SLC6A3*	2.28E−19	3.63E−16	2.13E−16	0.942168675	1.200859559	7.896317037	391/14754	415/18805	391
	5	GO:0050673	Epithelial cell proliferation	*RUNX2*	5.06E−19	6.44E−16	3.78E−16	0.930327869	1.185767627	7.935086435	454/14754	488/18805	454
	6	GO:0042391	Regulation of membrane potential	*CFTR*	7.69E−19	8.16E−16	4.79E−16	0.933045356	1.189231254	7.867901393	432/14754	463/18805	432
	7	GO:0045165	Cell fate commitment	*RUNX2*	1.26E−18	1.15E−15	6.74E−16	0.962711864	1.227043284	7.500934057	284/14754	295/18805	284
	8	GO:0072001	Renal system development		2.66E−18	2.12E−15	1.24E−15	0.952380952	1.213875817	7.549331907	320/14754	336/18805	320
	9	GO:0050878	Regulation of body fluid levels	*DGKA*/*SLC6A3*/*CFTR*	8.05E−18	5.69E−15	3.34E−15	0.940721649	1.199015224	7.559474533	365/14754	388/18805	365
	10	GO:0030098	Lymphocyte differentiation	*RUNX2*	1.29E−17	8.24E−15	4.83E−15	0.93287037	1.189008223	7.584578018	403/14754	432/18805	403
	11	GO:0001822	Kidney development		2.34E−17	1.31E−14	7.70E−15	0.950769231	1.211821566	7.351225766	309/14754	325/18805	309
	12	GO:0043010	Camera‐type eye development	*SLC6A3*	2.48E−17	1.31E−14	7.70E−15	0.942622951	1.201438565	7.426999062	345/14754	366/18805	345
	13	GO:0003012	Muscle system process		6.93E−17	3.39E−14	1.99E−14	0.925053533	1.179045119	7.477283245	432/14754	467/18805	432
	14	GO:0048568	Embryonic organ development	*RUNX2*/*LRIG1*	1.10E−16	4.99E−14	2.93E−14	0.925601751	1.179743861	7.423636337	423/14754	457/18805	423
	15	GO:0061138	Morphogenesis of a branching epithelium		1.50E−16	6.35E−14	3.72E−14	0.984293194	1.25455019	6.747907865	188/14754	191/18805	188
	16	GO:0042060	Wound healing	*DGKA*/*CHMP6*	3.61E−16	1.44E−13	8.43E−14	0.924444444	1.178268793	7.304715008	416/14754	450/18805	416
	17	GO:0072006	Nephron development		4.11E−16	1.51E−13	8.85E−14	0.99378882	1.266653027	6.484683705	160/14754	161/18805	160
	18	GO:0045785	Positive regulation of cell adhesion		4.27E−16	1.51E−13	8.85E−14	0.917835671	1.169845452	7.338484061	458/14754	499/18805	458
	19	GO:0001763	Morphogenesis of a branching structure		5.00E−16	1.67E−13	9.82E−14	0.975961538	1.243930916	6.751122017	203/14754	208/18805	203
	20	GO:0048732	Gland development	*SLC6A3*	1.03E−15	3.28E−13	1.92E−13	0.922394678	1.175656224	7.20585202	416/14754	451/18805	416
CC	1	GO:0097060	Synaptic membrane	*SLC6A3*	1.19E−20	9.21E−18	5.95E−18	0.933628319	1.207817464	8.247006885	422/15367	452/19880	422
	2	GO:0062023	Collagen‐containing extracellular matrix		3.09E−17	1.19E−14	7.70E−15	0.923611111	1.194858391	7.555838342	399/15367	432/19880	399
	3	GO:0045178	Basal part of cell		3.66E−16	9.41E−14	6.07E−14	0.940063091	1.216142009	7.158305581	298/15367	317/19880	298
	4	GO:0030139	Endocytic vesicle	*CFTR*/*HLA‐C*/*AP2A2*	7.44E−16	1.44E−13	9.27E−14	0.930167598	1.203340395	7.164108702	333/15367	358/19880	333
	5	GO:0009925	Basal plasma membrane		1.58E−14	2.43E−12	1.57E−12	0.936241611	1.211198231	6.778428691	279/15367	298/19880	279
	6	GO:0045177	Apical part of cell	*CFTR*/*PARD3*	3.03E−14	3.90E−12	2.52E−12	0.903966597	1.169444651	6.926960449	433/15367	479/19880	433
	7	GO:0016323	Basolateral plasma membrane		1.48E−13	1.63E−11	1.05E−11	0.939393939	1.21527634	6.497588204	248/15367	264/19880	248
	8	GO:0045211	Postsynaptic membrane	*SLC6A3*	1.99E−13	1.92E−11	1.24E−11	0.925233645	1.196957432	6.564636937	297/15367	321/19880	297
	9	GO:0016324	Apical plasma membrane	*CFTR*/*PARD3*	3.17E−13	2.72E−11	1.76E−11	0.907990315	1.174650059	6.618405831	375/15367	413/19880	375
	10	GO:0098984	Neuron‐to‐neuron synapse	*RGS1*	7.85E−13	6.05E−11	3.91E−11	0.905797101	1.17181274	6.518930048	375/15367	414/19880	375
	11	GO:0042734	Presynaptic membrane		1.19E−12	8.31E−11	5.37E−11	0.961325967	1.243649393	6.076334543	174/15367	181/19880	174
	12	GO:0030055	Cell‐substrate junction		1.93E−12	1.24E−10	7.99E−11	0.900921659	1.165505472	6.432817427	391/15367	434/19880	391
	13	GO:0005925	Focal adhesion		3.44E−12	2.04E−10	1.32E−10	0.900943396	1.165533593	6.357720565	382/15367	424/19880	382
	14	GO:0099572	Postsynaptic specialization	*RGS1*	5.15E−12	2.84E−10	1.83E−10	0.904040404	1.169540134	6.288400581	358/15367	396/19880	358
	15	GO:0032279	Asymmetric synapse	*RGS1*	5.72E−12	2.94E−10	1.90E−10	0.906914894	1.173258807	6.258734709	341/15367	376/19880	341
	16	GO:0009897	External side of plasma membrane	*HLA‐C*	6.45E−12	3.11E−10	2.01E−10	0.902743142	1.16786189	6.266140266	362/15367	401/19880	362
	17	GO:0031252	Cell leading edge		1.36E−11	6.17E−10	3.98E−10	0.896313364	1.159543807	6.201101212	389/15367	434/19880	389
	18	GO:0014069	Postsynaptic density	*RGS1*	7.70E−11	3.30E−09	2.13E−09	0.902777778	1.167906698	5.932510417	325/15367	360/19880	325
	19	GO:0030016	Myofibril		1.17E−10	4.75E−09	3.07E−09	0.926229508	1.198245762	5.749518277	226/15367	244/19880	226
	20	GO:0030136	Clathrin‐coated vesicle	*CFTR*/*AP2A2*/*RGS1*	1.26E−10	4.87E−09	3.15E−09	0.932432432	1.206270369	5.702992659	207/15367	222/19880	207
MF	1	GO:0046873	Metal ion transmembrane transporter activity	*SLC6A3*	3.02E−18	4.21E−15	3.00E−15	0.94	1.181194634	7.68116221	423/14833	450/18639	423
	2	GO:0001216	DNA‐binding transcription activator activity	*RUNX2*	4.16E−16	2.90E−13	2.07E−13	0.926680244	1.164457161	7.2905268	455/14833	491/18639	455
	3	GO:0001228	DNA‐binding transcription activator activity, RNA polymerase II‐specific	*RUNX2*	1.25E−15	5.81E−13	4.14E−13	0.925619835	1.163124661	7.178338548	448/14833	484/18639	448
	4	GO:0005516	Calmodulin binding	*RGS1*	3.90E−12	1.10E−09	7.83E−10	0.961352657	1.20802617	5.941507839	199/14833	207/18639	199
	5	GO:0022836	Gated channel activity	*CFTR*	3.94E−12	1.10E−09	7.83E−10	0.933962264	1.173607675	6.164355169	297/14833	318/18639	297
	6	GO:0035173	Histone kinase activity		2.46E−11	5.03E−09	3.58E−09	0.922222222	1.158855255	6.00836601	332/14833	360/18639	332
	7	GO:0140297	DNA‐binding transcription factor binding	*RUNX2*/*PADI2*	2.52E−11	5.03E−09	3.58E−09	0.905737705	1.138140975	6.104666026	442/14833	488/18639	442
	8	GO:0140996	Histone H3 kinase activity		3.87E−11	6.75E−09	4.81E−09	0.921568627	1.158033954	5.951856112	329/14833	357/18639	329
	9	GO:0141003	Histone H2AX kinase activity		6.09E−11	9.44E−09	6.72E−09	0.920903955	1.157198733	5.894988354	326/14833	354/18639	326
	10	GO:0015294	*Solute:monoatomic cation symporter activity*	*SLC6A3*	8.81E−11	1.23E−08	8.75E−09	0.99137931	1.245757363	5.241548754	115/14833	116/18639	115
	11	GO:0003779	Actin binding		1.04E−10	1.32E−08	9.43E−09	0.90744921	1.140291635	5.899638977	402/14833	443/18639	402
	12	GO:0008509	Monoatomic anion transmembrane transporter activity	*SLC6A3*/*CFTR*	1.41E−10	1.64E−08	1.17E−08	0.973154362	1.222856075	5.391739685	145/14833	149/18639	145
	13	GO:0005244	Voltage‐gated monoatomic ion channel activity		4.29E−10	4.60E−08	3.28E−08	0.959537572	1.205745352	5.367183322	166/14833	173/18639	166
	14	GO:0001217	DNA‐binding transcription repressor activity	*FOXK1*	7.12E−10	7.10E−08	5.05E−08	0.928057554	1.166187875	5.511290802	258/14833	278/18639	258
	15	GO:0005216	Monoatomic ion channel activity	*CFTR*	8.28E−10	7.70E−08	5.49E−08	0.901785714	1.133174943	5.632653029	404/14833	448/18639	404
	16	GO:0004674	Protein serine/threonine kinase activity	*CDKL1*	9.99E−10	8.71E−08	6.20E−08	0.903002309	1.134703704	5.598813201	391/14833	433/18639	391
	17	GO:0015276	Ligand‐gated monoatomic ion channel activity	*CFTR*	1.11E−09	9.11E−08	6.49E−08	0.951612903	1.195787292	5.297698533	177/14833	186/18639	177
	18	GO:0106310	Protein serine kinase activity	*CDKL1*	1.46E−09	1.13E−07	8.07E−08	0.911357341	1.145202553	5.499754173	329/14833	361/18639	329
	19	GO:0022832	Voltage‐gated channel activity		1.64E−09	1.20E−07	8.56E−08	0.954285714	1.19914592	5.225252738	167/14833	175/18639	167
	20	GO:0015103	Inorganic anion transmembrane transporter activity	*SLC6A3*/*CFTR*	1.95E−09	1.31E−07	9.31E−08	0.957575758	1.203280155	5.177692408	158/14833	165/18639	158

*Note:* Table 3 presents GO enrichment results based on 19,605 unique genes mapped from 164,400 significantly differentially methylated CpG sites (adjusted *p* < 0.05). Enrichment analysis was conducted using the clusterProfiler package in R, covering the three GO domains: Biological Process (BP), Cellular Component (CC), and Molecular Function (MF). Count, p.adjust, and other statistics reflect the full set of significant CpG‐associated genes. In the geneID column, only those genes among the previously identified top 20 differentially methylated genes that were also enriched in each GO term are listed. This highlights epigenetically altered genes with potential functional relevance in periodontitis while situating them within the broader methylation landscape.

### 2.10 Statistical Analysis

Earlier preprocessing and annotation reference generation steps were performed using R (version 4.3.0). All downstream statistical analyses, data integration, visualization, and interpretation were conducted using R (version 4.5.0) (Core Team R 2024), consistent with the scripts and README files provided in the Figshare repository. All downstream analyses, figures, and supplementary files reported in this manuscript, as were generated using this platform and corresponding scripts to ensure full reproducibility. Differential methylation analysis was performed using linear models with empirical Bayes moderation implemented in the limma package. CpG sites with an adjusted *p*‐value < 0.05 based on the Benjamini–Hochberg FDR were considered statistically significant. Unsupervised analyses, including hierarchical clustering and PCA, were applied to evaluate global methylation patterns. Directionality classification based on group‐wise average *β*‐values (≥ 0.5 as high‐methylation and < 0.5 as low‐methylation) was used solely as an exploratory approach to summarize group‐level methylation trends and was not intended to replace continuous differential methylation analysis.

GO enrichment analysis was performed using the clusterProfiler package with multiple‐testing correction applied to enriched terms.

## 3 Results

### 3.1 Global Methylation Differences Visualized by Volcano Plot

Volcano plot of all 484,331 CpG sites revealed a dense cluster of non‐significant sites around the origin and a smaller number of significantly differentially methylated CpG sites (adjusted *p* < 0.05) extending toward both tails.

The detection of many significant CpG sites reflects the genome‐wide scope and high sensitivity of the Illumina HumanMethylation450 BeadChip combined with the use of empirical Bayes–moderated linear models.

The majority of CpG sites did not exceed the threshold for significance, but a subset exhibited strong differential methylation. Vertical logFC threshold lines were intentionally omitted to focus on statistical significance (Figure 1).

### 3.2 Hierarchical Clustering Reveals Distinct Methylation Patterns

The heatmap of the 65 samples using the 10,000 randomly sampled significantly differentially methylated CpG sites showed distinct clustering patterns among control and periodontitis samples (Figure 2).

Samples within each group tended to cluster together, indicating group‐specific methylation profiles. This result suggests that periodontitis is associated with widespread changes in DNA methylation at these CpG loci.

### 3.3 Unsupervised Clustering and Ordination Separate Periodontitis and Control Samples

Hierarchical clustering of the 65 samples based on the 10,000 randomly sampled significantly differentially methylated CpG sites effectively grouped the samples by clinical status. Periodontitis and control samples formed distinct clusters, indicating consistent group‐level methylation differences (Figure 3A).

PCA further supported this separation, with the first two principal components, PC1 (31.6%) and PC2 (11.6%), accounting for a substantial proportion of variance and revealing distinct group distributions (Figure 3B).

### 3.4 Differential Methylation Analysis

A total of 164,400 significantly differentially methylated CpG sites were analyzed. Among these, methylation status changes between control and periodontitis groups were categorized into threshold‐crossing Control low → Periodontitis high and Control high → Periodontitis low groups based on group‐specific *β*‐values. The top 20 genes with the largest methylation changes were identified from these CpG sites (Table 1).

Cross‐tabulation of CpG sites by spatial relation to CpG islands and gene structural annotation revealed distinct distribution patterns. Most CpG sites with methylation changes were enriched in TSS200 and TSS1500 regions within or near CpG islands. In particular, gene body (Body) regions also contained substantial CpG sites with methylation changes associated with periodontitis, especially in OpenSea regions distant from CpG islands.

This comprehensive classification table (Table 2) integrates methylation changes, genomic context, and top gene associations, providing a detailed landscape of epigenetic alterations in periodontitis.

### 3.5 GO Enrichment Analysis in Periodontitis‐Associated Differentially Methylated Genes

GO enrichment analysis of the 19,605 genes associated with significantly differentially methylated CpG sites revealed distinct functional signatures (Table 3). In the BP category, the top enriched terms included epithelial and organ morphogenesis such as epithelial tube morphogenesis, epithelial cell proliferation, cell fate commitment, and embryonic organ development. In the CC category, enriched terms were predominantly localized to membrane‐ and vesicle‐related cellular components, including external side of plasma membrane, synaptic membrane, and clathrin‐coated vesicle. The MF category showed enrichment in transmembrane transporter activity, ion channel activity, and DNA‐binding transcription factor–related functions. These results indicate that DNA methylation alterations in periodontitis are linked to epigenetic regulation of epithelial structure, tissue remodeling, and membrane‐associated signaling, with additional implications for cell–cell communication and immune‐related cellular processes.

Furthermore, the CpG sites corresponding to both the top 20 genes and the enriched GO terms are presented in Table 3. Among the genes showing Control low → Periodontitis high threshold‐crossing shifts, eight genes, including *diacylglycerol kinase alpha (DGKA)*, *charged multivesicular body protein 6 (CHMP6)*, *RUNX family transcription factor 2 (RUNX2), cyclin dependent kinase like 1 (CDKL1)*, *solute carrier family 6 member 3 (SLC6A3)*, *CF transmembrane conductance regulator (CFTR)*, *major histocompatibility complex, class I, C (HLA‐C)*, and *leucine rich repeats and immunoglobulin like domains 1 (LRIG1)*, were found to be associated with enriched biological functions. In contrast, five genes, including *adaptor related protein complex 2 subunit alpha 2 (AP2A2)*, *par‐3 family cell polarity regulator (PARD3)*, *peptidyl arginine deiminase 2 (PADI2)*, *forkhead box K1 (FOXK1)*, and *regulator of G protein signaling 1 (RGS1)*, were identified in the Control high → Periodontitis low threshold‐crossing group.

In the Control low → Periodontitis high group, *DGKA* was associated with BP such as regulation of body fluid levels and wound healing. *CHMP6* was also linked to the BP term wound healing. *RUNX2* showed enrichment in several BP terms including epithelial cell proliferation, cell fate commitment, lymphocyte differentiation, and embryonic organ development, as well as MF terms such as DNA‐binding transcription activator activity, RNA polymerase II‐specific, and DNA‐binding transcription factor binding. *CDKL1* was related to MF terms including protein serine/threonine kinase activity and protein serine kinase activity. *SLC6A3* was associated with multiple BP terms such as sensory system development, visual system development, eye development, regulation of body fluid levels, camera‐type eye development, and gland development. It was also enriched in CC terms like synaptic membrane and postsynaptic membrane, and MF terms including metal ion transmembrane transporter activity, solute:monoatomic cation symporter activity, monoatomic anion transmembrane transporter activity, and inorganic anion transmembrane transporter activity. *CFTR* was linked to BP terms such as regulation of membrane potential and regulation of body fluid levels, CC terms including endocytic vesicle, apical part of cell, apical plasma membrane, and clathrin‐coated vesicle, as well as MF terms like gated channel activity, monoatomic anion transmembrane transporter activity, monoatomic ion channel activity, ligand‐gated monoatomic ion channel activity, and inorganic anion transmembrane transporter activity. *HLA‐C* was associated with CC terms endocytic vesicle and external side of plasma membrane, while *LRIG1* was linked to the BP term embryonic organ development.

In the Control high → Periodontitis low group, *AP2A2* was associated with the CC terms endocytic vesicle and clathrin‐coated vesicle. *PARD3* was linked to apical part of cell and apical plasma membrane. *PADI2* was related to the MF term DNA‐binding transcription factor binding, and *FOXK1* was enriched in the MF term DNA‐binding transcription repressor activity. *RGS1* showed associations with CC terms such as neuron‐to‐neuron synapse, postsynaptic specialization, asymmetric synapse, postsynaptic density, and clathrin‐coated vesicle, and was also linked to the MF term calmodulin binding.

## 4 Discussion

In this study, we performed an integrated epigenome‐wide DNA methylation analysis using three publicly available datasets to characterize the epigenetic landscape of periodontitis. The results revealed widespread methylation changes predominantly associated with epithelial structure, tissue remodeling, and membrane‐associated regulatory processes, with additional involvement of immune‐related pathways. All datasets were generated using the same Illumina HumanMethylation450 BeadChip platform and derived from gingival tissue samples collected at the same institution under comparable clinical and experimental protocols. These shared characteristics minimized platform‐ and site‐related heterogeneity and supported the validity of the integrated analysis. Although explicit batch‐effect correction was not applied, hierarchical clustering and PCA consistently separated control and periodontitis samples, suggesting that disease‐associated signals outweighed potential inter‐dataset variability.

The relatively large number of significant CpG sites identified should be interpreted in the context of epigenome‐wide association study design. The Illumina HumanMethylation450 BeadChip interrogates over 450,000 CpG sites, and empirical Bayes–moderated linear models implemented in limma are designed to provide stable inference in high‐dimensional settings with limited sample sizes (Ritchie et al. 2015; Phipson et al. 2016). Consistent with this, previous EWAS have shown that disease‐associated DNA methylation changes, particularly in immune‐mediated and chronic inflammatory conditions, can be distributed across many loci with modest effect sizes (Rakyan et al. 2011). Accordingly, the present analysis was primarily exploratory, and downstream biological interpretation focused on top‐ranked CpG‐associated genes with the largest absolute methylation differences.

Directionality classification based on a fixed *β*‐value threshold was applied as a complementary, exploratory framework to summarize group‐level methylation trends. However, this approach simplifies continuous methylation data and should therefore be interpreted with caution. Importantly, continuous differential methylation analysis using limma with empirical Bayes moderation was performed independently, and the direction‐based classification was not intended to replace linear modeling–based inference. Similar threshold‐based strategies have been employed in previous epigenetic studies to facilitate interpretation of disease‐associated methylation patterns (Alholle et al. 2015).

Integration of CpG island context with gene structural annotation revealed that a substantial proportion of differentially methylated CpG sites were located outside promoter‐associated CpG islands, particularly within gene body regions in OpenSea domains. Gene body methylation in such regions has been reported to modulate transcriptional fidelity, alternative splicing, and regulatory precision rather than directly suppressing gene expression (Jones 2012). This distribution contrasts with classical cancer epigenetic models that emphasize promoter CpG island hypermethylation (Esteller 2008; Jones and Baylin 2002).

GO enrichment analysis identified pathways related to epithelial function, tissue remodeling, and developmental processes, with additional links to immune‐related regulation. Notably, several enriched BP terms, such as lymphocyte differentiation, link these developmental pathways to immune‐related processes, highlighting the interplay between epithelial regulation and immune responses in chronic periodontal inflammation.

Hereafter, the terms “hypermethylated” and “hypomethylated” genes are used only to denote genes harboring CpG sites that crossed the *β* = 0.5 threshold between groups (Control low → Periodontitis high or Control high → Periodontitis low), as defined in the Section Material and Methods, 2, and do not refer to absolute methylation states.

Among hypermethylated genes, *RUNX2* showed gene body methylation in OpenSea regions and was enriched in GO terms related to cell fate commitment, epithelial proliferation, and immune‐related processes. *RUNX2* is a key transcription factor in osteogenesis and tissue regeneration (Kim and Adachi 2021; Komori 2017), and its altered methylation may contribute to impaired bone regeneration and epithelial repair in periodontitis, as suggested by previous experimental studies (An et al. 2015; Korinfskaya et al. 2021).

Other hypermethylated genes, including *CFTR*, *HLA‐C*, and *LRIG1*, were associated with epithelial barrier function and immune modulation. *CFTR* plays a central role in epithelial ion transport and barrier integrity and has been shown to be expressed in gingival tissues under inflammatory conditions (Ajonuma et al. 2010). Altered methylation of *CFTR* has also been reported in other chronic inflammatory diseases (Gaia‐Oltean et al. 2021), supporting its potential involvement in epithelial dysfunction during periodontitis.

Gene body and shore methylation have been reported to modulate transcriptional precision and regulatory control rather than acting as simple on–off switches (Jones 2012). Against this background, hypermethylated genes such as *HLA‐C* (Schaefer et al. 2008) and *LRIG1* (Ta et al. 2024), which are involved in antigen presentation and immune checkpoint regulation, respectively, may reflect epigenetic modulation of immune activity in chronically inflamed periodontal tissues.

Among genes showing Control high → Periodontitis low shifts, *RGS1* and *PADI2* were enriched in immune‐related pathways. *RGS1* has been implicated in immune cell migration and inflammatory signaling and has been reported as a disease‐associated gene in periodontitis and other inflammatory conditions (Zhang et al. 2022; Cheng et al. 2025). *PADI2* has been shown to be upregulated in periodontal tissues and to participate in inflammation‐associated epigenetic regulation through protein citrullination (Clancy et al. 2017; Engström et al. 2018). The observed hypomethylation of these genes may therefore reflect enhanced immune activation in periodontal inflammation.

Importantly, functional interpretations in this study should be considered with caution. DNA methylation changes do not necessarily translate directly into corresponding changes in gene expression, particularly in the absence of matched transcriptomic data. Functional implications were inferred based on genomic location, GO enrichment, and previously reported biological roles of the identified genes. These interpretations are therefore hypothesis‐generating rather than definitive. Future studies integrating DNA methylation with gene expression or other multi‐omics data will be necessary to validate the regulatory consequences of the methylation changes observed here.

A limitation of this study is the lack of harmonized, sample‐level demographic and clinical covariates across the public datasets, including age, sex, and quantitative measures of disease severity or inflammation. Although age and sex were reported for GSE53849, these variables were not consistently available for GSE59932 and GSE59939, and clinical indices (e.g., CAL, probing depth, and bleeding on probing) were not provided in a unified format across studies. Accordingly, covariate‐adjusted modeling could not be applied uniformly in the integrated analysis and should be addressed in future studies with standardized metadata. Therefore, the threshold‐based categorization was applied for interpretability and hypothesis generation, and is not intended to replace continuous differential methylation analysis based on linear modeling.

## 5 Conclusions

In conclusion, our comprehensive epigenome‐wide DNA methylation analysis revealed that periodontitis is associated with widespread, yet selective, methylation changes within the genomic regions interrogated by the Illumina HumanMethylation450 BeadChip, with a substantial proportion of significant sites observed in OpenSea regions and TSS‐proximal regions (TSS200 and TSS1500). This distribution pattern contrasts with the classical cancer epigenetic model where promoter CpG island hypermethylation is prevalent. Notably, the study identified multiple genes, such as *RUNX2*, *DGKA*, *CFTR*, *RGS1*, *PADI2*, and *FOXK1*, whose methylation status and GO enrichment suggest potential roles in tissue remodeling, immune regulation, and epithelial barrier function during periodontal inflammation.

These findings highlight the distinct epigenetic landscape of chronic inflammatory diseases like periodontitis and emphasize the importance of gene body and non‐CpG island methylation as regulatory mechanisms beyond traditional promoter silencing. Our results provide a valuable basis for future multi‐omics studies aiming to integrate DNA methylation with gene expression to further elucidate functional implications in periodontal disease pathogenesis. These findings not only provide new insights into the epigenetic landscape of periodontitis but also suggest that specific differentially methylated genes, such as *RUNX2*, *PADI2*, and *RGS1*, may serve as candidates for biomarker development, to be confirmed by future functional and clinical validation.

## Author Contributions

**Koki Yoshida:** data extraction, processing, formal analyses, data interpretation, draft writing and critical revision of the manuscript. **Gowri Sivaramakrishnan:** data extraction and processing. **Farah Asa'ad:** conceptualization, manuscript editing and critical revision of the manuscript.

## Funding

The authors received no specific funding for this work.

## Ethics Statement

The authors have nothing to report.

## Consent

The authors have nothing to report.

## Conflicts of Interest

The authors declare no conflicts of interest.

## Data Availability

The DNA methylation datasets analyzed in the current study are publicly available in the Gene Expression Omnibus (GEO) under accession numbers GSE53849, GSE59932, and GSE59939. All processed data, annotation results, R scripts, and supplementary datasets used to generate Figures 1–3 and Tables 1–3 are available on Figshare at: https://doi.org/10.6084/m9.figshare.29429705. The Figshare repository is publicly accessible and contains all supplementary datasets associated with this study.
